# What a Hug Does: A Qualitative Study of Chinese Immigrant Families’ Experiences with Inpatient Palliative Care Specialists

**DOI:** 10.1177/26892820251388866

**Published:** 2025-10-24

**Authors:** Zhimeng Jia, Allison Kurahashi, Ramona Mahtani, Siyi Fan, Lingsheng Li, Irene M. Yeh, Richard E. Leiter, Justin J. Sanders, James A. Tulsky, Rashmi K. Sharma

**Affiliations:** ^1^ Temmy Latner Centre for Palliative Care, Sinai Health, Toronto, Canada.; ^2^ Department of Family and Community Medicine, University of Toronto, Toronto, Canada.; ^3^ Department of Global Health and Social Medicine, Harvard Medical School, Boston, Massachusetts, USA.; ^4^ Department of Medicine, University of California, California, San Francisco, USA.; ^5^ Harvard Medical School, Boston, Massachusetts, USA.; ^6^ Department of Supportive Oncology, Dana-Farber Cancer Institute, Boston, Massachusetts, USA.; ^7^ Ariadne Labs, Boston, Massachusetts, USA.; ^8^ Department of Family Medicine, McGill University, Montreal, Canada.; ^9^ Cambia Palliative Care Center of Excellence, University of Washington, Seattle, Washington, USA.; ^10^ Department of Medicine, University of Washington, Seattle, Washington, USA.

**Keywords:** Asian, end-of-life, health disparities, immigrant, palliative care

## Abstract

**Background:**

Compared with non-Chinese adults in high-income countries, ethnically Chinese patients are more likely to encounter palliative care (PC) closer to death and in hospital settings. Yet, Chinese families’ experiences and perception of inpatient PC remain unknown.

**Objective:**

Identify barriers and facilitators to culturally respectful PC for Chinese immigrant inpatients and their caregivers.

**Design:**

Prospective, exploratory qualitative design involving phenomenological interviews.

**Setting/Subjects:**

We consecutively recruited (*n* = 15) Chinese immigrant patients and their caregivers (*n* = 14) referred to PC at one Canadian academic teaching hospital. We collected participant self-reported sociodemographics and Suinn-Lew acculturation level and conducted semi-structured interviews (*n* = 10) in Mandarin and/or English. The interviews were recorded, transcribed, translated, and thematically analyzed using Tan’s Health Communication framework.

**Results:**

Patients were older-aged (mean = 73.5 ± 16.2 years), 53.3% female, 60% college-educated, 66.7% nonreligious, and 93.3% diagnosed with cancer and had low acculturation (mean = 1.8 ± 0.9/5.0). Caregivers were middle-aged (mean = 50.6 ± 15.5 years), 78.6% children, 57.1% female, 85.7% college-educated, and 71.4% nonreligious and had moderate acculturation (mean = 2.5 ± 1.2/5.0). We identified four themes from post-consultation interviews: abandonment and alienation mark past experiences with serious illness care; emphasizing expertise and symptom relief may help overcome initial ambivalence toward PC; PC brokers competing priorities within the family unit; and PC alleviates time-related distress by addressing illness understanding.

**Conclusion:**

Chinese patients and caregivers may prefer a PC approach that is sensitive to historical mistrust, leverages expertise in symptom management to inspire confidence, and accommodates the information and care preferences of the family unit. Further research is needed to examine the impact of these PC strategies on clinical outcomes for Chinese families.

## Key Message

To enhance the delivery of palliative care to Chinese immigrant families, clinicians must ensure culturally and linguistically appropriate introductions to palliative care. Palliative care may also serve a mediating function, addressing intrafamilial conflicts and promoting illness understanding through attentive communication and presence.

## Introduction

The Asian population represents one of the fastest-growing and most ethnically diverse racial groups in North America and Europe.^1–4^ Population-based cohort studies consistently identify Asian patients, particularly Chinese immigrants, to be more likely to receive potentially burdensome life-sustaining treatments (e.g., mechanical ventilation, etc.) at the end-of-life compared with the general population.^5–7^ While the higher rates of invasive treatments at the end-of-life echo findings from other Chinese contexts,^8–10^ they contrast with Chinese community members’ preference against futile treatments.^11^ These conflicting findings signal unmet needs and culturally discordant care among Chinese immigrant patients at the end-of-life.^12^

Palliative care (PC) is “the active holistic care of individuals across all ages with serious health-related suffering due to severe illness and especially of those near the end of life.”^13^ In the hospital setting, randomized controlled trials have consistently demonstrated that specialist PC improves person-centered outcomes, including quality of life and symptom burden,^14^ and discharge to hospice.^15^ Recent research also identified that compared with the non-Chinese population, ethnically Chinese patients are more likely to first encounter specialist PC in hospital.^16^ Yet, gaps remain in our understanding of Chinese immigrant patients’ and families’ experiences and perceptions of specialist PC. Therefore, this qualitative study sought to identify elements of current PC practice that facilitate or impede culturally respectful care for this population.

## Methods

### Study design and setting

This single-site, prospective, observational cohort study explored the lived experiences of ethnically Chinese immigrant patients and their family members with the inpatient PC consult team at Mount Sinai Hospital (MSH), Toronto, Canada.^17^ The MSH is a 442-bed academic medical center, and its inpatient PC consult program is staffed by eight rotating physicians, two to four rotating learners, and one clinical nurse specialist. The study adhered to the Consolidated Criteria for Reporting Qualitative Research reporting guidelines and was approved by the Research Ethics Board of the MSH (ID:22-0093-E).^18^

### Participant recruitment

From September 1, 2022, to September 1, 2023, we screened every patient referred to the inpatient PC consult against a validated Chinese surname algorithm.^19^ We consecutively approached and recruited adult patients (≥18 years) who self-identified as ethnically Chinese (i.e., 华人), were born outside of North America, and spoke English or Mandarin. Patient’s designated surrogate decision-makers were also invited to participate dyadically. However, if eligible patients lacked decision-making capacity, then surrogates were eligible to participate independently (Table 1). A certified bilingual staff (ZJ, SF) was present for all recruitment and data collection. (Supplementary Fig. S1) We also professionally translated all study material into traditional and simplified Chinese. All participants that consented to the study received a CAN$50 gift card and an additional CAN$20 for completing the post-consultation interview.

**Table 1.** Eligibility Criteria for Patient and Caregiver Participants

Patient eligibility	Caregiver/proxy eligibility	Exclusion criteria
- Adult (age >18 years) - Self-identified as ethnically Chinese - Born outside of North America - Speaks English or Mandarin - Referred to the inpatient palliative care consult team - Aware of palliative care referral	- Adult (age >18 years) - Identified by patient as a key caregiver or by legal documentation - Speaks English or Mandarin	- Adults unable to consent (per clinical team assessment) - Infants, children, teenagers - Prisoners

### Data collection

On the day of consent (Day 0), we collected patients’ and caregivers’ sociodemographics through a structured questionnaire. If the patient lacked decision-making capacity or deferred decision-making to a designated family member, we collected these data from the caregiver. (Supplementary Fig. S1) After two days of interacting with the inpatient PC team (Day 2), we administered the Consultation and Relational Empathy Scale (CARE).^20,21^ (Supplementary Fig. S2) The CARE scale measures the cognitive, emotive, and behavioral domains of empathy using 10 items with Likert responses ranging from “1—poor” to “5—excellent.” The Cronbach’s alpha of the Chinese CARE is 0.96. (Supplementary Fig. S2)

We developed a semi-structured, phenomenological interview guide with the goal of understanding patients’ and caregivers’ lived experiences interacting with the PC consult team.^22^ The overall structure of the guide was informed by Tan’s Cultural Appropriateness for Health Communication framework,^23^ our prior work,^24–27^ and feedback from a multidisciplinary panel of qualitative research experts. Two authors (Z.J., S.F.) independently forward- and backward-translated the finalized guide into simplified Chinese. A certified third-party translator reviewed the final translations and resolved any ambiguous terms (Supplementary Table S1). Two authors (Z.J., S.F.) conducted interviews in English or Mandarin, and in-person, by phone, or via Zoom. To preserve ecological validity, interviews were audio-recorded and conducted individually or in dyads.^25^

### Data analysis

We descriptively analyzed participants’ sociodemographic, perceived clinician empathy, and transcribed recordings of interviews verbatim. The bilingual transcripts were uploaded to NVivo 12 software for analysis in their original language. We conducted a six-step directed thematic analysis to analyze the transcripts:^28^ (1) reading and rereading the transcripts to become familiar with primary data, (2) developing a codebook using deductive (i.e., Tan’s framework^23^ and our prior work^25^) and inductive approaches (i.e., open coding), (3) coding and recoding the transcripts using the finalized codebook, (4) comparing and combining similar codes into primary themes, (5) organizing the primary data (i.e., quotations) under each primary theme, and (6) contextualizing themes in relation to the essence of interacting with inpatient PC.

### Trustworthiness

We took the following steps to establish trustworthiness in our qualitative methodology.^28^ First, two authors (Z.J., R.S.) held monthly peer debriefing sessions to establish an audit trail of the development of themes. Second, the principal investigator (Z.J.) provided a self-critical account of the genesis, conduct, and future directions of the research (Supplementary Table S2). Third, we addressed credibility of the findings by member checking the findings at research conferences and clinical staff meetings. Finally, we rigorously documented the study setting, study procedure, and study population to establish transferability and confirmability. We collectively approached this research with an anti-racist^29^ and critical realist lens.^30^

## Results

### Participants

**FIG. 1.**
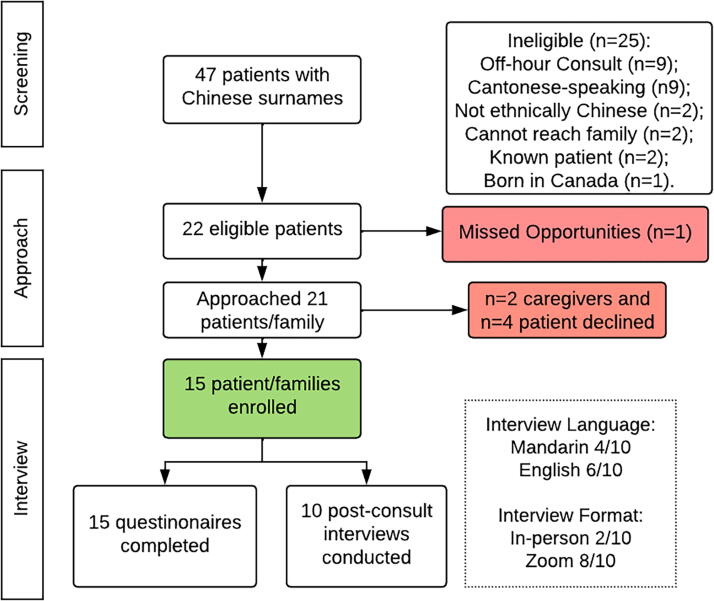
Recruitment flow chart.

A total of 47 patients screened positive for an ethnically Chinese surname. We approached 21 patients and recruited 15 patients (71.4%) and 14 accompanying caregivers. (Fig. 1) Participating patients were older-aged (73.5 ± 16.2 years), 53.3% female, 60% partnered/married, 60% college-educated, 66.7% nonreligious, and 100% insured. Regarding acculturation, many patients (46.7%) identified as Asian and had lived in Canada for an average of 33.9 ± 19.3 years. Clinically, most patients’ primary diagnosis was cancer (93.3%), the median palliative performance status was 40 (interquartile range [IQR] 40–60), and the most common disposition setting was home (66.7%). Participating caregivers were middle-aged (50.6 ± 15.5 years), 57.1% female, 71.4% married, 85.7% college educated, 71.4% nonreligious, and 78.6% adult children. Caregivers were more acculturated than patients, with most caregivers identifying as Asian-Canadian (71.4%) and having lived in Canada for an average of 32.3 ± 17.1 years (Table 2).

**Table 2.** Demographic and Clinical Characteristics of Participants

	Patient (*n* = 15)	Caregiver (*n* = 14)
Age, mean years (SD)	73.5 (16.2)	50.6 (15.5)
Female, *n* (%)	8 (53.3%)	8 (57.1%)
Partnered/married, *n* (%)	9 (60%)	10 (71.4%)
College education, *n* (%)	9 (60%)	12 (85.7%)
Provincial health insurance, *n* (%)	15 (100%)	—
Caregiver relationship to patient, *n* (%)		
Spouse	—	2 (14.3%)
Child	—	11 (78.6%)
Sibling	—	1 (7.1%)
Born in greater China region, *n* (%)	15 (100%)	10 (71.4%)
Years in Canada, mean (SD)	33.9 (19.3)	32.3 (17.1)
Suinn-Lew Self Identity, *n* (%)		
Asian	7 (46.7%)	3 (21.4%)
Asian-Canadian (Asian First)	4 (26.7%)	4 (28.6%)
Asian-Canadian (Blend)	4 (26.7%)	5 (35.7%)
Asian-Canadian (Canadian First)	—	1 (7.1%)
Canadian	—	1 (7.1%)
Religious or spiritual affiliation, *n* (%)		
Secular and nonreligious	10 (66.7%)	10 (71.4%)
Christian	2 (13.3%)	2 (14.3%)
Buddhism	2 (13.3%)	2 (14.3%)
Primary cancer diagnosis, *n* (%)	14 (93.3%)	—
Palliative performance status, median [Interquartile Range]	40 [40–60]	—
Palliative care consultant is bilingual and ethnically Chinese, *n* (%)	7 (46.7%)	
Code status at the time of consult		—
Full code	3 (20%)	—
Do not resuscitate	7 (46.7%)	—
Unknown	5 (33.3%)	—
Final disposition		—
Home	10 (66.7%)	—
Palliative care unit or hospice	3 (20%)	—
Rehabilitation facility	1 (6.7%)	—
Hospital death	1 (6.7%)	—

SD, standard deviation.

### Consultation and relational empathy

**FIG. 2.**
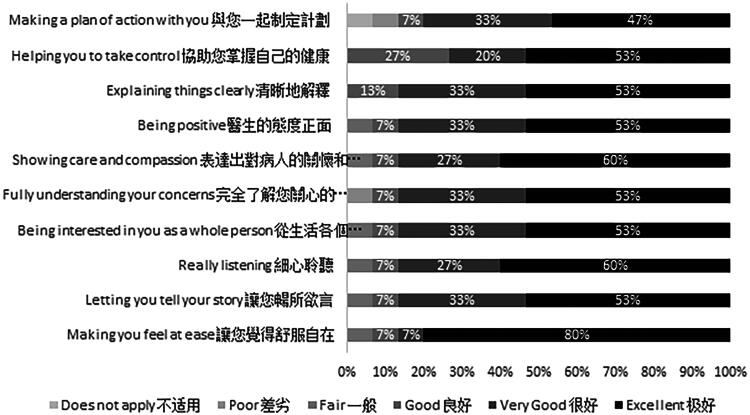
Ratings of the inpatient palliative care team’s relational empathy.

Participants had favorable ratings of clinicians’ relational empathy. Eleven out of the 15 participating families (73.3%) gave at least one top-box rating (i.e., “excellent”) in the 10-item CARE survey. Among the six participating families that gave top-box ratings across all 10 items, five (83.3%) received consultations from bilingual and ethnically Chinese consultants. The item with the highest proportion of top-box ratings in the CARE survey was “Making you feel at ease” (80.0%). The item with the lowest proportion of top-box ratings was “Making a plan of action with you” (46.7%). (Fig. 2)

### Qualitative themes

We conducted 10 post-consultation interviews representing 10 caregivers and one patient. Most interviews were conducted in English (60%) and virtually (80%). The median time from consult to interview was 8 days (IQR 6–167 days). The median duration of the interviews was 29 minutes (IQR 26–34 minutes). Hereunder, we illustrate four major themes with representative quotes.

### Theme 1: Abandonment and alienation mark past experiences with serious illness care

Reflecting on their serious illness journey, many participants expressed feeling abandoned by the health care system. The sense of abandonment was most pronounced during care transitions and when multiple providers were involved:

“This side refers you, but the other side doesn’t accept you, and you feel like no one wants you. It was a very scary time.” (ID 15)

“This the [Social Worker], told us, ‘Your mom have choice. Choice is going to [Institution] or get out of here. Go to home.’ That was, we are devastated… they forced her going to those facility, without give a good explanation.” (ID 19)

Immigrant families’ sense of alienation was exacerbated by limited social support and language barriers:

“I think Chinese people enjoy company and when they don’t have the same race of people or don’t have anyone to talk to about how to deal with the treatments or how to deal with the life-threatening illnesses, it becomes lonely in the sense that they have nowhere to go and they have nowhere to think.” (ID 29)

“A lot of older generation from mainland China or from Hong Kong, they don’t speak English. Even if they do, they would communicate better with their own native language.” (ID 13)

The resulting unmet needs, when combined with language barriers, could intensify perceived malintent during medical care:

“I hear my dad calling out to his nurse, but the person just ignored him and kept walking away… I’m not sure if they’re just shirking their responsibility.” (ID 28)

“So for example, at the beginning when our doctor told us to go home, right?… Why did they give up on treatment? Why did they tell us to wait at home for him to die?” (ID 4)

### Theme 2: Emphasis on expertise and symptom relief may help overcome initial ambivalence toward palliative care

When participants reflected on the recommendation to involve PC, they described a torrent of emotions marked by uncertainty and fear:

“Please explain to me, what is PC? Is it no treatment? Just to make him feel comfortable, or? I didn’t quite understand…” (ID 28)

“When I went on their website, I didn’t like the way it said, “End of life and death.” That really turned me off because it gave me the chills.” (ID 30)

Reflecting on their own experiences, participants endorsed introductions of PC that emphasized expertise and symptom relief:

“So, when you guys come, I always tell my dad: “The doctor to help you with pain is here… because they are more professional and are more knowledgeable. The current medications are not optimized, they know better medications.” (ID 15)

“To your point, why would you need to go to another team for pain management? I think that needs a little bit more education than, like, specialists who help you increase, through pain management or like, increased healing, stuff like that.” (ID 10)

### Theme 3: Palliative care brokers competing priorities within the family unit

Many participants described the intergenerational and intrafamilial challenges around decision making:

“With our generation, I always ask: “Is this the right treatment?”… Whereas their generation, they would just take what the doctor said.” (ID 30)

“But in many cases, if we only consider the family’s feelings, then the patient is constantly being tortured, because s/he can’t express his/her own ideas. But if we only consider a patient’s feelings, like helping them feel comfortable, and die quickly, but maybe the family will not accept this… Because the most difficult choices people have to make are not for themselves, but on behalf of others.” (ID 4)

Several participants described how, with time, they were able to appreciate PC’s role beyond the physical to the socioemotional aspects. A few families noted that having ethnically concordant clinicians may facilitate this process.

“Then I realized that [PC] goes way beyond that and goes more towards daily caregiving needs so that the patient doesn’t have to go through the health care system like an average patient and improve their quality of life…” (ID 26)

“When I spoke to [PC physician], it gave me a sense of security, knowing that someone of the same race is taking care of my mom emotionally and mentally checking up on her health and making sure that the care process that she is given is going to be followed through and that he will be there for her when it’s needed.” (ID 29)

When patients and caregivers perceive the PC team as benevolent and as having a clear understanding of the family’s care preferences, then clinicians are better situated to mediate expectations across cultural contexts and to align health care resources with sacred family values.

“The benefits of having a PC team, in my opinion, actually, I think the best about it is that it can act as a mediator and take care of two issues at the same time. The current Canadian system of healthcare or our healthcare in China, they can only solve one problem, that is, ‘how can I save the patient and make the patient feel happy?’ But they may not have any idea what the family thinks, right?… You’ll find that in PC, they would use their actions and their language to demonstrate what a hug does, and you’ll feel that way at the end, you’ll feel warm inside.” (ID 4)

### Theme 4: Palliative care alleviates time-related distress by addressing illness understanding

Many participants described how they saw health care professionals as responsible for delivering and helping families understand prognostic information.

“I think that as a PC doctor, (s)he is responsible for patient’s understanding of their illness and illness trajectory, to make appropriate preparations, and accept death.” (ID 20)

“I think that conversation was really important to clarify what our plan was moving forward because I think that for a while, we were still in limbo… juggling between extending life and just keeping quality of life as well.” (ID 26)

Several participants expressed how PC played an important role in providing anticipatory guidance and supporting the dignity of the patient by minimizing burdensome interventions.

“I think the most important thing is that they helped give us a care plan. They helped us see the big picture as to what to expect and come up with a plan to keep my father comfortable for as long as possible.” (ID 26)

“How should I say it, in the last period of life, rather than being in constant struggle, and finally losing some really good time with my father, it was better to accept the current facts and through medical treatment, reduce my father’s pain to the minimum, so [I] could stay with him for a longer period of time, maybe this was the best for the last farewell.” (ID 4)

## Discussion

The rapid growth of the Chinese immigrant population in North America signals a pressing need to identify ways to deliver culturally respectful serious illness care to Chinese immigrant patients and their families. To this end, this qualitative study is the first to examine the experiences of Chinese immigrant inpatients and families receiving specialty PC. Our findings revealed four key themes that shape Chinese immigrant families’ experience with PC: (1) abandonment and alienation mark past experiences with serious illness care, (2) emphasis on expertise and symptom relief may help overcome initial ambivalence toward PC, (3) PC brokers competing priorities within the family unit, and (4) PC alleviates time-related distress by addressing illness understanding. Taken together, our results highlight PC clinicians’ critical roles in ensuring linguistically appropriate introduction of PC, mediating intrafamilial discussions of optimal caring, and interpreting time-based information.

Our study confirms and expands upon existing literature describing Chinese immigrant patients’ and families’ experiences with PC.^25,31^ Previous studies in the UK, Australia, and Canada described Chinese immigrant families’ limited knowledge of and misunderstandings toward PC.^31^ Our findings add to existing research by uncovering participants’ historical negative experiences with care and preferences for an introduction of PC centered on expert relief of burdensome symptoms (e.g., physical, psychosocial, etc.). Especially during language discordant contexts, palliative care clinicians should be cognizant of the evolving terminology surrounding hospice and PC, which ranges from time-based (临终关怀, lin zhong guan huai) to emphasizing appeasement (姑息治疗, gu xi zhi liao) to comfort-focused (安宁疗护, an ning liao hu) to relief-centered (缓和医疗, huan he yi liao).^32,33^ A recent Delphi study with 61 Chinese palliative care professionals identified 缓和医疗 (huan he yi liao) and 安宁疗护 (an ning liao hu) as the optimal translations of palliative care and hospice care, respectively.^34^

Participants rated specialist PC clinicians’ favorably in the CARE survey, especially in the domains of “really listening,” “showing care and compassion,” and “making you feel at ease.” These findings contrast with CARE ratings of interactions in non-PC settings,^20,35,36^ suggesting that PC clinicians may be more adept at demonstrating empathy.^37^ Our qualitative findings echoed this, as participants described relational conflicts rooted in intergenerational differences and tensions with medical care (i.e., acculturation stress),^38^ which threatened interpersonal harmony. PC clinicians were seen as playing a mediating role, navigating these intrafamilial dynamics through presence, listening, and attuning to socioemotional aspects of care.^24^

The lower CARE ratings of “making a plan of action with you” contrast with participants’ appreciation for PC clinicians’ guidance in illness understanding discussions and end-of-life preparation.^24,25,39–41^ This discrepancy may reflect the temporal sequence of data collection, where the survey captured impressions from the first three days, whereas the interviews elicited reflections beyond this period. The qualitative findings suggest that PC clinicians may be uniquely positioned to identify and accommodate the sociocultural and information needs of Chinese immigrant families.^41,42^ Future research should link clinician behaviors during naturally occurring discussions^43–45^ with patient satisfaction^46,47^ and preparedness^48–50^ to identify effective strategies that promote felicitous intercultural goals-of-care discussions with immigrant families.

### Limitations

This single-site qualitative study of Chinese immigrant patients’ and caregivers’ inpatient PC experiences has several limitations. First, restricting recruitment to patients with fluency in English or Mandarin limits the transferability of our findings to other linguistic communities of the global Chinese diaspora. Second, the delay in interviews from time of consultation, as a result of patients’ rapidly declining status, may have introduced recall bias into caregivers’ recollection of their experiences in hospital. Finally, patients’ rapidly declining health limited their participation in post-consultation interviews. Thus, further triangulation of our predominant caregiver interview findings with patients and Chinese families at other institutions may improve the transferability of our results.

## Conclusion

This qualitative study explores Chinese immigrant families’ experience with inpatient PC specialists. To improve PC engagement in this population, PC clinicians may consider an approach that is sensitive to historical mistrust, leverages expertise in symptom management to inspire confidence, and accommodates the information and care preferences of the family unit. Further research is needed to triangulate Chinese families’ PC experiences across clinical contexts and to elucidate optimal communication strategies that maintain familial harmony while promoting shared illness understanding.

## Data Availability

The data that support the findings of this study are available from the corresponding author, Z.J., upon reasonable request.

## Data Access, Responsibility, and Analysis

Dr. Jia had full access to all the data in the study and take responsibility for the integrity of the data and accuracy of the data analysis.

## Prior Presentations

The major findings of this study were presented at the American Academy of Hospice and Palliative Medicine State of Science conference on March 24^th^, 2024; the 2024 Kathleen M. Foley Palliative Care Research Symposium on September 27^th^, 2024; and the Hospice Waterloo Community Advance Care Planning conference on March 2^nd^, 2025.

## Abbreviations Used

CARE: Consultation and Relational Empathy Scale
MSH: Mount Sinai Hospital
PC: Palliative Care
SF: Siyi Fan
ZJ: Zhimeng Jia
