# Supplementary material for: What a Hug Does: A Qualitative Study of Chinese Immigrant Families’ Experiences with Inpatient Palliative Care Specialists
Source: Palliat Med Rep. 2025 Oct 24;6(1):494–502. doi: 10.1177/26892820251388866 (PMC12670663; doi:10.1177/26892820251388866)
Supplement: Supplementary Data [file 26892820251388866_supplementary_data.docx]

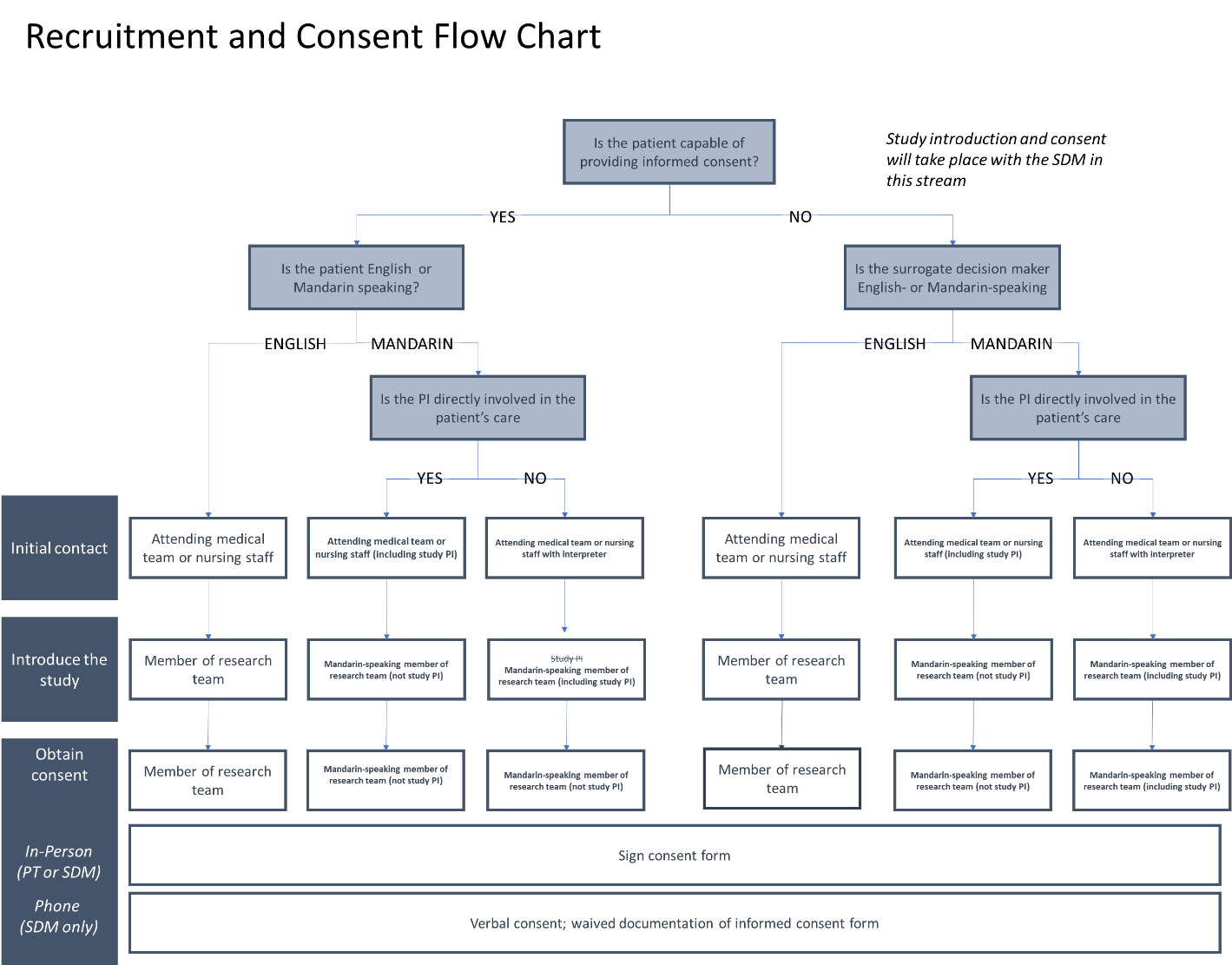


Figure S1: Study introduction and consent process stratified by language proficiency and the role of the principal investigator (PI). When non-PI palliative care physicians served as staff consultants, PI acted as a researcher, implementing study activities. In instances where language interpretation was required, PI provided clinical interpretation support. When PI was serving in the role of the staff physician, a member of the study team (SF) conducted all research-related activities, ensuring a clear separation of roles. This division of responsibilities was documented in the consent process, and participants were provided written confirmation of our respective roles.


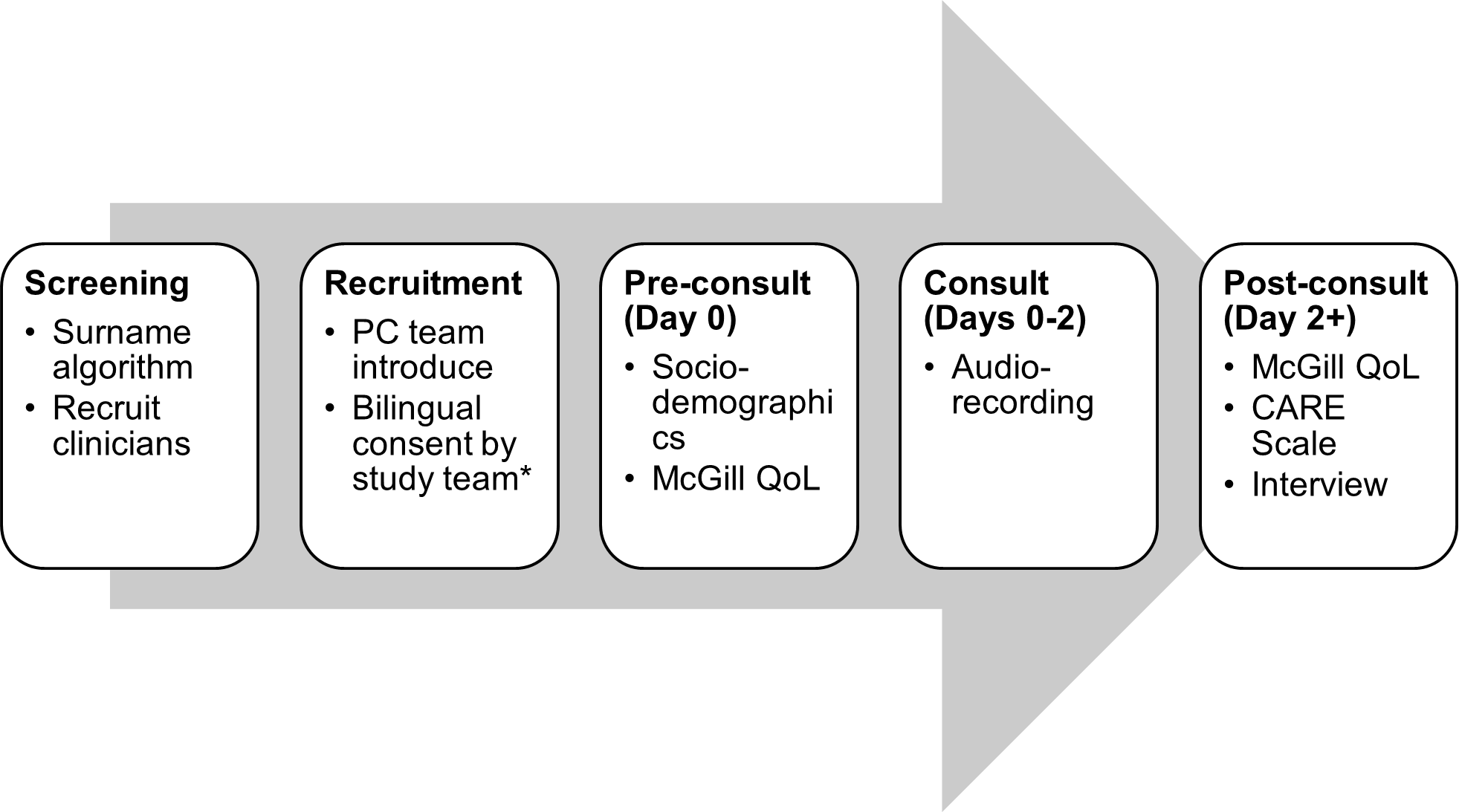


Figure S2: Overview of study instruments

Table S1: Bilingual Interview Guide

| **INTRODUCTION （介绍）**  Thank you so much for your willingness to participate in this study. We know that your time is precious and appreciate your willingness to share your thoughts and personal experiences with us.  十分感谢你参与我们的这项研究。我们知道您的时间是宝贵的，所以很珍惜接下来与您探讨您的想法和经历。  The goal of this study is to help palliative care clinicians understand aspects of doctor-patient communication that are most helpful for patients and their families from the Chinese community. We hope the results of the study will allow more Chinese patients and families to receive care and make treatment decisions that are aligned with their values and preferences.  这项研究的目的是让缓和医疗医师更加了解如何与华裔患者和家属沟通。我们希望通过这项研究帮助更多的华人得到以人为本，符合文化背景的医疗服务。  All the questions I will ask you concern your personal views. There are no right or wrong answers. I also want to remind you that you don’t have to answer any question that you don’t want to. Your answers will be kept confidential and anonymous. Nothing you say will be attributed to you. Nothing will be communicated with your attending doctors. Your answers will not affect your treatment, prescriptions, nor ongoing relationship with your clinical team.  在我们后面的对话中，我只在乎您的个人观点。答案并无对错。如果哪个问题您不想回答，您不必回答。您的回答将完全匿名，我们会对谈话内容负有保密责任。您所说的一切不会被追溯到您身上。我们也不会把您说的话反应给您的医疗团队。这样您的答案不会影响治疗方案以及您与医疗团队的关系。  Finally, I would also like to record our conversation, so I can be more present and engaged during our conversation while still accurately capturing your ideas and suggestions. Would that be ok? What other questions do you have before we start?  最后，我希望对这场对话录音，这样既能准确的记录您的想法以用于后期研究，也可以让我把注意力放在对话上。您同意录音吗？在我们开始之前您还有什么问题吗？ |
| --- |
| **Background （文化背景）**   - I’d like to start by learning about you and your family’s experience with medical care. **What is it like receiving medical care in Canada?**   我想首先了解一下您和您的家人的医疗经历。可以谈谈您在加拿大的就医的经历吗？   - - Can you share an experience that may be unique to Chinese patients and family?   有没有哪些经历是华人患者独有的？   - - In order to best care for Chinese individuals, what aspects should the Canadian medical system consider?   以便更好的照顾华人患者，加拿大的医疗系统应该考虑到哪些方面？   - Now I’d like you to think back to when you first came to Mount Sinai Hospital or (Date of hospitalization), **what has happened since then**?   下面，我希望您可以回想来到西奈山医院的第一天（住院的日子），从那以后都发生了什么？   - - How would you compare the care here with previous medical care experiences? What has been done well? What could be improved?   您会怎样对比在这里和您以前在其他医院的医疗经历？ 有什么是西奈山医院做的好的地方？ 有什么地方可以改进呢？ |
| **Experience with Palliative Care （缓和医疗经历）**  Next, I’d like to talk about you and your family’s experience of palliative care. We can stop at anytime if you feel the conversation becomes too difficult or for any other reason.  接下来，我想谈谈您和您的家人在缓和医疗方面的经历。如果您觉得谈话变得太困难或出于任何其他原因，我们可以随时停止。   - I’d like you to pause for a moment and notice anything that comes to mind when you hear the words “Palliative Care.” **What images, sensations or feelings come to mind**?   我希望我们可以稍微暂停片刻。当您听到“缓和医疗”这几个字的时候，您脑海中会浮现出什么画面或者身心会有什么反应吗？   - Now I’d like you to take me back to the first time you learned that palliative care became involved in your care. **What was on your mind at that time**? What were your impressions of Palliative Care at that time?   下面，我想让您带我回到您第一次知道缓和医疗被邀请来帮您会诊。当时您在想什么？当时您对缓和医疗有什么样的印象？   - Take some time now and think about a **memorable conversation** with the palliative care team (Drs ____ and ____). The memory may be a positive or a challenging one. How would you describe this conversation? How did you feel during this conversation? What made this conversation memorable?   下面，希望您可以花一点时间回想一次让您难忘的与缓和医疗医生的谈话。能为我描述一下吗？在谈话中您感觉如何？是什么让这次谈话难忘？   - Sometimes doctors and patients could **misunderstand each other** because they come from different backgrounds or have different expectations. What role did language or communication play in your interaction with the palliative care team? When did the communication work well? In what ways could the team have done better?   有时候，不同的背景和期望会让医生,病人和照顾者之间产生误解。您有类似的担忧吗？方便讲一讲您的经历吗？有哪些方面医疗团队可以改进的？   - Throughout the course of the hospitalization, did your family’s **understanding of palliative care change**? If so, in what ways? What, if anything, surprised you and your family about palliative care?   在您的住院期间，您和您的家人对缓和医疗的理解是否发生了变化？如果有，在哪些方面呢？缓和医疗有没有让您和您的家人感到惊讶的地方？   - Thinking back, in what ways, if any, was palliative care **helpful** to you and your family? What was the **most important role** that palliative care played during you and your family’s hospital stay?   您觉得缓和医疗在哪些方面帮助到了您或者您的家人？在您的住院期间，缓和医疗发挥的最重要的作用是什么呢？   - Given what you know now, how would you **explain palliative care** to your family and friends? What meaning does palliative care have within your family? How do you think palliative care should be introduced to Chinese patients and families?   鉴于您现在所知道的，您会怎样与您的家人和朋友解释缓和医疗？缓和医疗对您的家庭有什么意义？您认为应该怎样向华裔患者和家属介绍缓和医疗？ |
| **Closing question （结束）**   - Thank you so much for your time. Before we end, is there anything important that I haven’t asked you? What else would you like to talk about that we haven’t covered?   再次感谢您的时间。在我们结束这次访谈之前，还有什么我们应该考虑的重要因素吗？还有什么您希望讨论的话题吗？   - What is one piece of advice you would give other Chinese patients or families in a similar situation?   您会给处于和您类似情况的其他华人患者或者家属什么建议呢？ |

Table S2: Study principal investigator’s reflexive stance

| Researcher | Reflexive account |
| --- | --- |
| ZJ | As a male, first-generation Chinese Canadian in my mid-thirties, my personal and professional experiences have deeply shaped my interest in this research. My role as a staff palliative care consultant and clinician investigator within the same institution where the study was conducted afforded me institutional power and resources to explore how Asian immigrant families experience palliative care. My motivation for this research stems from personal experiences as a bereaved son of my immigrant father in the mid-2000s and as a bereaved son-in-law of my immigrant father-in-law in 2023. Their experiences of dying—one in hospice, the other in an ICU—have fueled my commitment to understanding how palliative care can better support immigrant families in navigating serious illness while ensuring they feel respected and cared for.  At the outset, I assumed that aspects of palliative care—how it is introduced, practiced, and experienced—might feel foreign or even jarring to immigrant families. However, through this study, I learned that the sense of abandonment and alienation often predates palliative care involvement. I also discovered that after experiencing palliative care, which requires time and rapport building, many immigrant patients and families developed a more positive perception of its intentions and benefits.  This study has influenced my approach to future research. Given time constraints and our desire to understand modifiable clinician behaviors to accommodate patients’ and families’ care preferences, future studies will focus on consultations referred for disposition planning or goals-of-care discussions. Additionally, this work has prompted reflection on the culture of palliative care consultation teams. Unlike some other consult services, which may defer communication interventions to the primary medical team, our team is highly engaged in direct communication with patients and families. Understanding these differing team dynamics will be important for future research on optimizing palliative care delivery in diverse healthcare settings. |
